# Understanding HIV epidemiology: a comprehensive analysis of demographic, clinical, and virological profiles of patients with HIV at a tertiary care center

**DOI:** 10.1016/j.ijregi.2026.100978

**Published:** 2026-08-13

**Authors:** Hassan A. Abdou, Faryal Khamis, Mohab Sherif, Jalila Al Naamani, Kawther Al Bahrani, Asmahan Alyaaqoubi, Salah T. Al Awaidy

**Affiliations:** 1 Faculty of Medicine, Capital University (formerly Helwan University), Cairo, Egypt; 2 Infectious Diseases Unit, Department of Medicine, Royal Hospital, Ministry of Health, P.O.Box 393 PC 100, Muscat, Oman; 3 Department of Nursing, Royal Hospital, Ministry of Health, P.O.Box 393 PC 100, Muscat, Oma; 4 School of Medical Sciences, University of Manchester, Manchester, P.O.Box 393 PC 100, Muscat, Oman, United Kingdom; 5 Pulmonary Unit, Department of Medicine, Royal Hospital, Ministry of health, P.O.Box 393 PC 100, Muscat, Oman; 6 Freelance Public Health Senior Consultant, P.O.Box 393 PC 100, Muscat, Oman

**Keywords:** HIV, Antiretroviral therapy, Viral suppression, CD4 count, Co-infection, Oman

## Abstract

**Objectives:**

Updated, center-specific cohort data on people living with human immunodeficiency virus (HIV) (PLWH) from the Arabian Peninsula remain limited. We describe the demographic, clinical, and laboratory profiles of PLWH receiving antiretroviral therapy (ART) at Oman’s main national referral center.

**Methods:**

We conducted a single-center retrospective cohort study of 454 PLWH between January 2010 and December 2023 at the Royal Hospital, Oman. Demographic, clinical, virological and immunological variables were extracted from the electronic medical records. Non-parametric tests, Spearman and Kendall correlations, the Kruskal-Wallis test, and a binomial logistic regression model were used to characterize the cohort and identify independent predictors of virological non-suppression.

**Results:**

The cohort was predominantly male (64.3%) and Omani (96.5%), with a mean age at diagnosis of 30.3 years (SD 13.8 years). Heterosexual transmission was the most common route. At presentation, 66.9% of patients were classified as World Health Organization clinical stage 1 and 15.5% as stage 4, followed by stage 2 (9.0%) and stage 3 (8.5%); the median initial cluster of differentiation 4 (CD4) count was 254 cells/µL, and the median baseline viral load was 71,141 copies/mL. The most frequent ART regimen was tenofovir disoproxil fumarate/emtricitabine/efavirenz (28.6%). In multivariable logistic regression (*n* = 410; McFadden *R²* = 0.149), older age at HIV diagnosis was independently associated with lower odds of virological non-suppression (odds ratio 1.094 per year, 95% CI 1.053-1.137; *P* < 0.001), while an initial CD4 count ≥200 cells/µL was associated with higher odds of non-suppression (odds ratio 0.326, 95% CI 0.114-0.934; *P* = 0.037).

**Conclusion:**

This cohort reflects a predominantly male, heterosexually acquired HIV epidemic at this center, with a substantial burden of advanced disease at presentation and clinically relevant co-infection rates. Younger age at diagnosis was an independent risk factor for virological non-suppression, a finding that may support targeted retention and adherence interventions in younger PLWH.

## Highlights

• This 13-year retrospective cohort study examined 454 people living with human immunodeficiency virus (HIV) who were receiving antiretroviral therapy at Oman’s main national referral center.
• The cohort was predominantly male and Omani; most patients presented at an early, asymptomatic World Health Organization clinical stage, but 15.5% already had advanced, acquired immune deficiency syndrome-defining disease at diagnosis.
• Older age at HIV diagnosis independently predicted a lower likelihood of virological non-suppression, whereas a higher initial cluster of differentiation 4 count was, counterintuitively, associated with a higher likelihood of non-suppression.
• These findings underscore the need to sustain robust national and regional HIV care cascades and to provide targeted retention and adherence support for younger people living with HIV.

## Introduction

Human immunodeficiency virus (HIV) infection remains a major public health priority requiring lifelong antiretroviral therapy (ART). Progress against the epidemic has not been uniform across regions: the Middle East and North Africa (MENA) region is one of the few regions in which HIV incidence continues to rise rather than decline, with new infections increasing by approximately 31% between 2010 and 2023 and an acquired immune deficiency syndrome (AIDS)-related mortality rate that remains disproportionately high, without the corresponding reductions seen in sub-Saharan Africa, Eastern Europe, or Southeast Asia. This divergence emphasizes the urgent need for region-specific data to guide targeted public health responses [1,2].

In Oman, the first case of HIV/AIDS was documented in 1984, and the National AIDS Program was established in 1996 [3]. Oman is classified by the World Bank as a high-income country, and HIV testing, treatment, and follow-up monitoring are provided free of charge to citizens through the country’s nationalized, government-funded health system. Oman has since built a comprehensive, publicly funded HIV care infrastructure, including 14 treatment centers offering free ART, HIV genotyping, viral load (VL), and cluster of differentiation 4 (CD4) count monitoring nationwide. Oman adopted the “treat-all” policy, irrespective of CD4 cell count, in December 2015, aligning with World Health Organization (WHO) recommendations [4]. National surveillance data indicate that annual new HIV diagnoses in Oman have exceeded 130 cases per year in recent years, with predominantly heterosexual transmission being the principal driver of the epidemic and males being disproportionately affected [4].

Prior single-center studies from the Royal Hospital, Muscat, the primary national HIV referral facility, have characterized the epidemiological and clinical profile of people living with HIV (PLWH) in Oman. A study of 326 patients with HIV seen over 27 years (1989-2016) reported a mean age of 36 years, male predominance (60.4%), and heterosexual transmission as the leading mode of acquisition (58.9%) [5]. More recently, a 30-year retrospective study from Sohar Hospital in North Batinah (1995-2024) confirmed the shift toward predominant heterosexual transmission (56%) and male predominance (70.6%), and identified a lower CD4 count as a significant predictor of mortality [6]. At the national level, Elgalib et al. [7] examined the HIV cascade of care in Oman in 2019, demonstrating that, of an estimated 2440 PLWH, only 55% were virally suppressed, highlighting considerable gaps at the population level, particularly among those aged 13-24 years and those receiving care outside Muscat Governorate.

Cohort studies are of particular importance in the management of HIV because they allow the characterization of treatment outcomes, identification of predictors of virological failure, and tracking of disease progression over time. In the Gulf Cooperation Council (GCC) region, HIV prevalence remains below 0.01%, yet the 95-95-95 targets remain unmet [8,9]. Data from Bahrain, Oman, Qatar, and the United Arab Emirates indicate that, by 2021, viral suppression among those on ART ranged from 55% to 92% [8,9]. Regional cohort data from Saudi Arabia and comparisons across GCC nations underscore the importance of local datasets to tailor ART strategies and improve patient outcomes [10,11].

Despite existing research, no study has characterized PLWH at this center beyond 2016, and none spans the dolutegravir-transition era or the post-“treat-all” policy period. This 13-year cohort (2010-2023) addresses that gap by providing contemporary data from Oman’s primary national referral center during a period of fundamental change in first-line ART. The primary aim of this study is to describe the demographic, clinical, and laboratory characteristics of PLWH receiving ART at the Royal Hospital, Muscat, over this period. The secondary aim is to identify independent predictors of virological non-suppression at last follow-up. These findings are intended to inform clinical practice and public health policy and contribute to the broader evidence base for the MENA region.

## Methods

### *Study design and setting*

A retrospective cohort study was conducted among patients enrolled in the HIV/AIDS database established at the Royal Hospital, Muscat, Oman, a Ministry of Health tertiary hospital that serves as the primary national referral center for HIV care. Data were extracted from electronic medical records spanning January 2010 to December 2023 using a standardized data extraction sheet.

### *Data collection*

Data collected encompassed the following: (i) demographic variables, including age, sex, marital status, nationality, and governorate of residence; (ii) HIV-related variables, including mode of transmission, categorized as heterosexual contact, men who have sex with men (MSM), intravenous drug use, vertical (mother-to-child) transmission, blood transfusion, or unknown/undisclosed, WHO clinical stage at presentation (classified according to the WHO 2007 guidelines) [12], and comorbidities, including diabetes mellitus and hypertension; (iii) co-infection screening, including hepatitis B surface antigen (HBsAg), hepatitis C virus (HCV) immunoglobulin G (IgG) antibody, and syphilis serology assessed using both non-treponemal tests (Rapid Plasma Reagin and Venereal Disease Research Laboratory tests) and confirmatory treponemal testing, where indicated; (iv) tuberculosis (TB) co-infection status, defined according to Oman Ministry of Health and WHO criteria encompassing both clinical/radiological diagnosis and microbiological confirmation (culture or nucleic acid amplification testing) [13]; (v) clinical variables, including body mass index at initial presentation and at 6-month intervals thereafter, ART regimens comprising nucleoside/nucleotide reverse transcriptase inhibitor backbone combinations with either non-nucleoside reverse transcriptase inhibitors, protease inhibitors, or integrase strand transfer inhibitors as the third agent, and treatment-related adverse effects, including nephrotoxicity, hepatotoxicity, lipodystrophy, neuropsychiatric effects, and gastrointestinal intolerance; and (vi) laboratory variables, including baseline complete blood count, renal function tests, liver function tests, CD4 cell count, and HIV VL at diagnosis and following ART initiation, with CD4 count and VL monitored at 6-month intervals throughout the follow-up period.

### *Statistical analysis*

All statistical analyses were performed using jamovi (version 2.6.44; The jamovi project, 2024), which operates on R (version 4.4; R Core Team, 2024). A two-tailed significance level of *P* < 0.05 was applied throughout. No imputation was performed for missing data; all analyses were conducted on complete cases.

Continuous variables were summarized as mean ± SD and median (with range); categorical variables as frequencies and percentages. The normality of all continuous variables was formally assessed using the Shapiro-Wilk test; as all variables significantly deviated from normality (all *P* ≤ 0.002), non-parametric methods were applied throughout.

Bivariate associations between continuous and ordinal variables were assessed using Spearman’s rank correlation coefficient (*ρ*) and Kendall’s tau-b. Pearson’s *r* was additionally computed where both variables were continuous and numeric; it is not reported (denoted as “not applicable”) for binary/categorical variables for which computation is undefined. All three coefficients are presented to allow comparison of monotonic and linear associations.

The Kruskal-Wallis test, a non-parametric analog of one-way analysis of variance, was used to compare VL change (VL Change) across groups defined by initial CD4 count categories. Two separate tests were conducted: one using the binary CD4 threshold of 200 cells/µL (First_CD4_200) and another using 500 cells/µL (First_CD4_500). Effect size was quantified using epsilon squared (*ε²*), interpreted as small (<0.04), medium (0.04-0.16), or large (>0.16).

A binomial logistic regression model was constructed to identify independent predictors of virological non-suppression at last follow-up. The binary outcome variable, Last_VL_1000, was defined as VL ≥1000 copies/mL at the most recent measurement (1 = unsuppressed; 0 = suppressed [<1000 copies/mL]). The following predictor variables were entered into the model simultaneously: age at HIV diagnosis (continuous, years), VL change from baseline (VL Change, continuous), initial CD4 count below 200 cells/µL (First_CD4_200: 0 = CD4 <200, 1 = CD4 ≥200), and initial CD4 count below 500 cells/µL (First_CD4_500: 0 = CD4 <500, 1 = CD4 ≥500).

Model fit was evaluated using the McFadden pseudo-*R²*, Akaike information criterion, and the omnibus likelihood-ratio chi-square test. Multicollinearity was assessed using the variance inflation factor (VIF) and tolerance statistics, with VIF > 10 considered indicative of problematic multicollinearity. The predictive performance of the model was assessed using sensitivity at a classification cutoff of 0.5. Odds ratios (ORs) and 95% CI were derived by exponentiating the log-odds coefficients.

### *Ethical considerations*

This study was a retrospective cohort analysis of data routinely collected and extracted from the electronic medical records of the Royal Hospital. No patients were contacted or interviewed. Ethical approval was granted by the Scientific Research Committee, Royal Hospital (SRC#26929). The principles of the Declaration of Helsinki were adhered to in the design, execution, and reporting of this investigation.

## Results

### *Socio-demographic and clinical characteristics of the study population*

A total of 454 patients with HIV receiving ART during the study period were included in this retrospective cohort study. The cohort was predominantly male (*n* = 292, 64.3%; *P* = 0.383) and of Omani nationality (*n* = 438, 96.5%; *P* = 0.615). Most patients presented at an early, asymptomatic WHO clinical stage 1, although a clinically meaningful minority presented with advanced (stage 4) disease at diagnosis (WHO clinical staging available for *n* = 387; *P* = 0.476). Patients were geographically concentrated in Muscat Governorate and North Batinah Governorate, broadly reflecting the largest population concentrations nationally and established health care infrastructure (*P* = 0.441; Table 1).

**Table 1:** Socio-demographic and clinical characteristics of the study population overall and by virological suppression status at last follow-up (*N* = 454).

Characteristic	Overall (*N* = 454)	Virologically suppressed (*n* = 421)	Virologically unsuppressed (*n* = 30)	*P*-value
Total patients, *n*	454	421	30	—
Age at current follow-up, years, median (mean ± SD)	39.8 (39.7 ± 13.7)	40.2 (40.4 ± 13.4)	31.3 (30.5 ± 14.7)	<0.001
Age at HIV diagnosis, years, median (mean ± SD)	30.0 (30.3 ± 13.8)	30.0 (31.1 ± 13.5)	20.0 (18.5 ± 14.0)	<0.001
Marital status, *n* (%)				<0.001
Married	250 (55.6%)	240 (58.1%)	9 (30.0%)	
Single/unmarried	113 (25.3%)	103 (24.9%)	9 (30.0%)	
Child	53 (11.9%)	42 (10.2%)	11 (36.7%)	
Divorced	19 (4.3%)	18 (4.4%)	0 (0.0%)	
Widowed	11 (2.5%)	10 (2.4%)	1 (3.3%)	

Data are from a retrospective single-center cohort of 454 people living with HIV receiving antiretroviral therapy at the Royal Hospital, Muscat, Oman’s primary national HIV referral center, between January 2010 and December 2023. Virological suppression was defined as a last available HIV-1 viral load <1000 copies/mL (*n* = 421), and non-suppression as ≥1000 copies/mL (*n* = 30) at last follow-up; suppression status was unavailable for 3 patients, who were therefore excluded from the group-specific columns and *P-*value calculations. Between-group comparisons used the Mann-Whitney *U* test for continuous variables and the chi-square test for the categorical variable shown. This table presents only the characteristics that showed a statistically significant bivariate association with virological suppression status; sex, nationality, WHO clinical stage, hepatitis C virus IgG serostatus, hepatitis B surface antigen serostatus, tuberculosis co-infection, governorate of residence, first CD4 count, and first viral load were also assessed but showed no significant association (all *P* > 0.05) and are reported in the socio-demographic and clinical characteristics of the study population section instead. Marital status was available for *n* = 446.

Abbreviations: HIV, human immunodeficiency virus.

Age at last follow-up and at the time of HIV diagnosis, together with marital status, was significantly associated with viral status. Marital status was significantly associated with virological suppression. Additionally, younger patients at the time of diagnosis and at the last follow-up were less likely to achieve virological suppression. The remaining demographic and clinical characteristics of the cohort, none of which showed a significant bivariate association with suppression status, are summarized below (Table 1).

HCV IgG seropositivity was identified in 16 patients (4.0%) and HBsAg positivity in 24 patients (6.0%); neither showed a statistically significant bivariate association with virological suppression status (*P* = 0.573 and *P* = 1.000, respectively). TB co-infection was documented in a clinically meaningful minority of screened patients (23 of 147 screened, 15.6%; *P* = 0.167), comprising both active (*n* = 3) and latent (*n* = 9) disease, with the remainder unclassified. Screening was performed using QuantiFERON-TB Gold interferon-gamma release assay, supplemented by sputum smear microscopy and bronchoscopy where clinically indicated.

The mode of HIV transmission was documented in 187 patients (41.2%); among those with a recorded mode, heterosexual contact was predominant (*n* = 146, 78.1% of documented cases), followed by MSM/bisexual exposure (*n* = 20, 10.7%), vertical transmission (*n* = 13, 7.0%), intravenous drug use (*n* = 6, 3.2%), and blood transfusion (*n* = 2, 1.1%). The mode of transmission was not recorded for the remaining 267 patients (58.8%), likely reflecting social and legal stigma surrounding disclosure.

The most frequently prescribed ART regimens were all based on tenofovir disoproxil fumarate/emtricitabine (TDF/FTC), combined with efavirenz (*n* = 130, 28.6%), dolutegravir (*n* = 60, 13.2%), or atazanavir/ritonavir (*n* = 48, 10.6%). Baseline first CD4 count and first VL(Table 2) likewise did not differ significantly between suppression groups (*P* = 0.224 and *P* = 0.998, respectively).

**Table 2:** Descriptive statistics for continuous variables.

Variable	*N*	Missing	Mean	Median	SD	Min	Max	S-W *P*
Age at diagnosis (years)	413	41	30.3	30.0	13.8	0.07	68.0	<0.001
Current age (years)	454	0	39.7	39.8	13.7	4.1	73.6	0.002
First CD4 (cells/µL)	421	33	333	254	332	0	1939	<0.001
First VL (copies/mL)	419	35	502,603	71,141	1,400,000	0	10,000,000	<0.001
VL Change (copies/mL)	451	3	454,710	48,200	1,360,000	−1,040,000	10,000,000	<0.001

Data are from the same 454-patient cohort described in Table 1. *N* = number of patients with a non-missing value for the variable; Missing = number of patients with no recorded value. VL Change = last viral load minus first viral load (copies/mL); a negative value indicates a decrease in viral load between the first and last measurement. S-W p = *P*-value for the Shapiro-Wilk test of normality; *P* < 0.05 indicates significant departure from a normal distribution, supporting the use of non-parametric statistical methods for all continuous variables in this study.

Abbreviations: S-W, Shapiro-Wilk; VL, viral load.

### Descriptive statistics of continuous variables

The descriptive statistics for the continuous variables are summarized in Table 2. The mean age at diagnosis was 30.3 years (median 30.0 years; SD 13.8), whereas the mean age at last follow-up was 39.7 years. The mean CD4 count at initial presentation was 333 cells/µL (median 254; SD 332). The mean VL at initial presentation was 502,603 copies/mL. The change in VL between the first and last measurement showed a mean decrease of 454,710 copies/mL (median 48,200; SD 1,360,000 copies/mL), with a broad range of change from a decrease of 1,040,000 in some patients to an increase of 10,000,000 copies/mL in others.

The descriptive statistics for the principal continuous variables, including measures of central tendency, dispersion, and normality, indicate that both VL-related variables (first VL and VL change) showed substantial positive skewness (skewness = 4.90 and 5.06, respectively), consistent with the right-skewed distribution typical of HIV VL data.

Shapiro-Wilk testing confirmed that all continuous variables significantly deviated from normality (Table 2), justifying the use of non-parametric statistical methods throughout the analysis.

### Correlation analysis

A comprehensive correlation matrix was generated using Spearman’s *ρ*, Kendall’s tau-b, and Pearson’s *r*; the pairwise correlations most relevant to the study aims are reported below. VL Change demonstrated a near-perfect positive correlation with first VL (Spearman’s *ρ* = 0.971, *P* < 0.001; Pearson’s *r* = 0.998), indicating that these two variables are essentially collinear, as patients with higher baseline VLs showed correspondingly greater absolute changes.

Both binary CD4 threshold variables, representing CD4 ≥200 cells/µL (First CD4 200) and CD4 ≥500 cells/µL (First CD4 500), for which a value of 1 indicates a CD4 count at or above the respective threshold, demonstrated strong negative correlations with the continuous first CD4 count (Spearman’s *ρ* = −0.858 and −0.716, respectively; both *P* < 0.001). These findings validate the internal consistency of the categorical CD4 thresholds in relation to the continuous CD4 measure.

Regarding the primary outcome, VL change was significantly negatively correlated with virological non-suppression, defined as a Last VL ≥1000 copies/mL (Spearman’s *ρ* = −0.185, *p* < 0.001), suggesting that greater VL reduction is associated with viral suppression (VL <1000 copies/mL) at last follow-up. The continuous Last VL was strongly correlated with virological non-suppression status (Spearman’s *ρ* = 0.500, *P* < 0.001), confirming the validity of the binary outcome measure.

Sex showed no significant correlation with virological non-suppression (Spearman’s *ρ* = 0.050, *P* = 0.286), nor did HCV IgG, HBsAg, or marital status (all *P* > 0.05).

### Kruskal-Wallis tests

Table 3 presents the results of Kruskal-Wallis tests comparing VL change, defined as Last VL minus first VL, across categories of initial CD4 count based on complete cases.

**Table 3:** Kruskal-Wallis tests: VL change by initial CD4 category.

Comparison	*χ²*	df	*P*-value	Effect size (*ε²*)
VL Change by initial CD4 count (cutoff: <200 vs ≥200 cells/µL)	12.5	1	<0.001	0.028
VL Change by initial CD4 count (cutoff: <500 vs ≥500 cells/µL)	7.50	1	0.006	0.017

The Kruskal-Wallis test is a non-parametric analog of one-way analysis of variance, used here to compare viral load change (last viral load minus first viral load) across groups defined by initial CD4 count using complete cases. First CD4 200 groups: CD4 <200 vs ≥200 cells/µL; First CD4 500 groups: CD4 <500 vs ≥500 cells/µL. Significance threshold: *P* < 0.05.

Abbreviations: CD4, cluster of differentiation 4; df, degrees of freedom; VL, viral load; ε², epsilon squared; χ², chi-square test statistic.

For the first comparison, using a CD4 cutoff of <200 versus ≥200 cells/µL, the Kruskal-Wallis test revealed a statistically significant difference in VL change between the two groups (*χ²* = 12.5, df = 1, *P* < 0.001). The corresponding effect size (*ε²* = 0.028) was small, indicating that although the difference in VL change between CD4 categories was statistically significant, the magnitude of this association was small. For the second comparison, using a CD4 cutoff of <500 versus ≥500 cells/µL, the Kruskal-Wallis test also showed a statistically significant difference in VL change between groups (*χ²* = 7.5, df = 1, *P* = 0.006). As with the first comparison, the effect size (*ε²* = 0.017) was small.

### Binomial logistic regression

A binomial logistic regression model was fitted on a complete-case sample to identify independent predictors of last VL status (Last VL: 0 = suppressed [<1000 copies/mL], 1 = unsuppressed [≥1000 copies/mL]). The overall model was statistically significant, showed an acceptable fit to the data, and was free of problematic multicollinearity among predictors (Table 4).

**Table 4:** Binomial Logistic Regression: Predictors of Viral Non-Suppression (Last_VL_1000 = 1)

**Predictor**	**B**	**95% CI Lower**	**95% CI Upper**	**SE**	**Z**	**p**	**OR (95% CI)**
**Intercept**	1.406	0.418	2.394	0.504	2.79	0.005	—
**Age at diagnosis (years)**	0.090	0.052	0.128	0.020	4.60	<.001	1.094 (1.053–1.137)
**First CD4 ≥200 vs <200**	−1.122	−2.175	−0.068	0.538	−2.09	0.037	0.326 (0.114–0.934)

Predictors were entered simultaneously in a binomial logistic regression model fitted on a complete-case sample (N = 410). B = log-odds regression coefficient; SE = standard error; Z = Wald test statistic; OR = odds ratio (exp[B]); 95% CI = 95% confidence interval. Outcome variable: Last_VL_1000, defined as HIV-1 viral load ≥1000 copies/mL at last follow-up (1 = virologically unsuppressed) versus <1000 copies/mL (0 = virologically suppressed). Viral load change from baseline and initial CD4 count ≥500 cells/µL (versus <500 cells/µL) were also entered into the model as covariates but did not reach statistical significance and are omitted from this table for clarity; coefficients for these two covariates are reported in the text. Overall model fit: likelihood ratio χ²(4) = 28.9, p < .001; McFadden pseudo-R² = 0.149; Akaike Information Criterion (AIC) = 174.8; Variance Inflation Factor (VIF) range 1.01–1.31 and tolerance range 0.76–0.99, indicating no problematic multicollinearity among predictors; model sensitivity = 0.997 at a 0.5 classification threshold. Bold rows indicate statistically significant predictors (p < 0.05). Coefficients incorporate correction of two miscoded First_CD4_200 records identified in the source data; the correction had a negligible effect on all coefficients (a minor refinement to the First CD4 ≥200 row and the overall model fit statistics shown here).

Results of the omnibus likelihood-ratio tests indicated that age at HIV diagnosis (*χ²*(1) = 26.4, *P* < 0.001) and initial CD4 count ≥200 cells/µL (*χ²*(1) = 4.85, *P* = 0.028) were significant predictors of viral suppression. In contrast, VL change (*χ²*(1) = 1.57, *P* = 0.210) and initial CD4 ≥500 cells/µL (*χ²*(1) = 0.55, *P* = 0.458) did not reach significance at the omnibus level. These findings are consistent with the coefficient-level (Wald) tests reported below. Coefficient-level results are presented in Table 4. Age at HIV diagnosis was an independent, statistically significant predictor of viral status, such that older age at diagnosis was associated with higher odds of viral suppression at last follow-up; that is, older age at diagnosis was a protective factor against virological non-suppression. Initial CD4 count ≥200 cells/µL (versus <200 cells/µL) was also a statistically significant independent predictor of viral suppression, with patients presenting with CD4 ≥200 cells/µL having lower odds of viral suppression than those presenting with CD4 <200 cells/µL. This counterintuitive finding suggests that patients with more advanced disease (CD4 <200 cells/µL) were more likely to achieve viral suppression at last follow-up, possibly reflecting more intensive clinical monitoring, greater treatment urgency, and potentially higher treatment adherence in this subgroup.

Neither VL change from baseline (*B* = 2.50 × 10⁻⁷, *P* = 0.304) nor an initial CD4 count ≥500 cells/µL (*B* = −0.504, *P* = 0.458) was an independent predictor of virological outcome in the multivariable model. Both variables were included in the model as covariates but, given their lack of statistical significance, were omitted from Table 4 for simplicity of presentation.

## Discussion

This 13-year retrospective cohort included 454 PLWH receiving ART at Oman’s national referral center. While two-thirds presented at WHO stage 1, 15.5% presented with advanced AIDS-defining illness. A substantial burden of co-infection was documented in 25.6% of the cohort. Older age at diagnosis was independently associated with higher odds of virological non-suppression at follow-up, whereas a baseline CD4 count ≥200 cells/µL was associated with lower odds of non-suppression, consistent with better baseline immune status supporting virological control. These results extend the available evidence base for Oman’s HIV epidemic into the treat-all and dolutegravir-transition eras and have direct implications for optimizing the care cascade at both the center and national levels.

The mean age at HIV diagnosis of 30.3 years and the male predominance (64.3%) observed in our cohort are consistent with findings from prior studies. Khamis et al. [5], in their 27-year analysis at the same center, reported a mean cohort age of 36 years and a male predominance of 60.4%. Similarly, the 30-year Sohar Hospital cohort reported a male predominance of 70.6% and a mean age at diagnosis of 32.8 years [6]. At the national level, Elgalib et al. [4] demonstrated that more than two-thirds of annual new HIV cases in Oman were among males, with the majority aged 25-49 years.

The high proportion of Omani citizens (96.5%) reflects the structure of the national HIV program, which primarily serves Omani citizens at public treatment centers. This is comparable to the composition reported in prior Omani cohort studies [14]. Across the MENA region, male predominance in HIV cohorts is a well-established pattern, with a male-to-female ratio averaging approximately 3:1 in most Arab countries. This disparity is likely multifactorial, reflecting the greater social freedom and mobility afforded to men in the region, which increases opportunity for sexual exposure outside stable partnerships. Additionally, it may partly reflect differential access to and uptake of HIV testing and diagnosis, with women, particularly those outside antenatal care settings, facing greater structural and stigma-related barriers to health care-seeking. Underreporting of female cases, driven by social stigma and legal consequences associated with HIV disclosure in many Arab societies, may further contribute to the observed gender imbalance [15].

Two-thirds of patients (66.9%) presented at WHO clinical stage 1 (asymptomatic disease), suggesting that a substantial proportion were diagnosed through screening programs (e.g., antenatal, pre-employment, or blood donation screening) rather than symptomatic presentation. However, the second most prevalent stage was WHO clinical stage 4 (15.5%), indicating that a clinically significant minority still present with advanced, AIDS-defining illness. This bimodal distribution has been observed in other regional cohorts [5,6]. In Saudi Arabia, Al-Harthi et al. [11] reported that up to 50% of patients at a major referral center had AIDS at diagnosis. Late presentation with advanced immunodeficiency remains a major challenge across the MENA region, associated with higher early mortality and suboptimal immune recovery. The drivers of late diagnosis are likely multifactorial, including deeply entrenched social stigma surrounding HIV, low risk perception among individuals who may not identify themselves as belonging to traditionally recognized at-risk groups, cultural and religious taboos that discourage open discussion of sexual behavior and HIV testing, and a prevailing mistrust of confidentiality within health care settings. Collectively, these factors deter timely presentation and voluntary HIV testing. Addressing these barriers through community-based, stigma-free testing initiatives and strengthened confidentiality frameworks will be essential to reduce the burden of late-stage diagnosis in Oman and the broader MENA region.

The median baseline CD4 count of 254 cells/µL in our cohort reflects a mixed pattern of presentation, with some patients retaining relative immune competence and others presenting with profound immunosuppression. The median CD4 threshold of 200 cells/µL has well-established prognostic significance; the landmark COHERE collaboration demonstrated that a higher CD4 cell count among patients receiving combination ART with a suppressed VL is consistently associated with a reduced risk of AIDS events and death [16]. Conversely, patients with persistently low CD4 counts despite viral suppression have a 2.6-fold greater risk of mortality than those with higher counts [17].

The geographic distribution of patients, with 43.0% from Muscat Governorate (the capital of Oman), reflects the concentration of the population and health care infrastructure in the capital. However, the meaningful representation of patients from North Batinah (18.1%) and South Batinah (9.3%) is consistent with national surveillance data showing a steady increase in the proportion of new HIV cases diagnosed outside Muscat Governorate [4]. These findings have important implications for service planning. The 2021 cascade-of-care analysis by Elgalib et al. [7] found that patients receiving HIV care outside Muscat experienced the greatest attrition in transitioning from linkage to retention in care. This underscores the need for continued decentralization of HIV services and targeted outreach in non-capital governorates.

Heterosexual contact was the predominant mode of HIV transmission in our cohort, consistent with findings across Oman and the broader GCC region [3]. National surveillance in Oman has consistently demonstrated heterosexual transmission as the main epidemic driver, although a notable proportion of cases are classified as having an unknown or undisclosed mode, likely reflecting underreporting in the context of social and legal stigma surrounding same-sex behavior and intravenous drug use in the region [4,6]. The MENA region has historically reported heterosexual transmission as the most common mode in most countries, although HIV epidemics among MSM appear to be emerging in several nations [15].

HBsAg positivity (6.0%) and HCV IgG positivity (4.0%) in our cohort are broadly consistent with hepatitis B virus (HBV) and HCV co-infection rates reported in other MENA cohorts [18]. HBV co-infection has significant implications for ART selection, given the activity of tenofovir-based regimens against both HIV and HBV, and is associated with accelerated liver fibrosis and increased risk of hepatocellular carcinoma [19,20]. The prevalence of TB co-infection (15.6% of screened patients) is clinically meaningful; HIV/TB co-infection remains a leading cause of AIDS-related mortality globally, and integrated diagnosis and treatment pathways are critical [21].

The most frequently prescribed ART regimen was TDF/FTC/efavirenz, reflecting the period (2010-2023) during which this regimen was the WHO-recommended first-line treatment for most patients [22]. The second most common regimen was TDF/FTC/dolutegravir, consistent with the global and regional transition toward integrase strand transfer inhibitor-based regimens following updated WHO guidelines that recommend dolutegravir as the preferred first-line therapy from 2019 onward [23]. A 3-year observational cohort from Saudi Arabia reported bictegravir/emtricitabine/tenofovir as the most prescribed ART regimen during the study period, reflecting more recent prescribing practices [10].

Our multivariable logistic regression identified two independent predictors of virological outcome at last follow-up: age at diagnosis and baseline CD4 count category. Older age at diagnosis was associated with higher odds of viral suppression (OR = 1.094 per year, 95% CI: 1.053-1.137, *P* < 0.001). This finding aligns with established evidence that older patients receiving ART demonstrate higher rates of virological suppression, likely attributable to greater treatment adherence, better tolerability of modern ART regimens, and reduced engagement in high-risk behaviors. A meta-analysis by Zhang et al. demonstrated that older patients had higher rates of viral suppression (risk ratio: 1.04, 95% CI: 1.01-1.08) after 36 months of ART initiation, albeit with poorer immunological recovery [24]. The Canadian Observational Cohort similarly reported that patients aged >60 years were more likely to achieve viral suppression (adjusted hazard ratio = 1.20, 95% CI: 1.05-1.37) than those aged ≤30 [25]. In contrast, a higher proportion of younger adults were in the unsuppressed group, an observation that is consistent with other studies and reinforces the case for enhanced adherence support in younger PLWH. Consistent with this age-related pattern, marital status was also significantly associated with virological suppression status. This could be due largely to spousal social and emotional support, facilitated disclosure, and buffering against HIV-related stigma [26].

The observation that presenting with a CD4 ≥200 cells/µL was paradoxically associated with lower odds of viral suppression at last follow-up (OR = 0.326, 95% CI: 0.114-0.934, *P* = 0.037) compared with a CD4 <200 cells/µL warrants careful interpretation. This counterintuitive finding may reflect differences in the intensity of clinical follow-up and treatment urgency: patients presenting with advanced immunosuppression (CD4 <200 cells/µL) are more likely to receive frequent monitoring, adherence counseling, and prompt ART optimization, all of which contribute to sustained virological suppression [27]. Alternative explanations cannot be excluded, including regression to the mean, a healthy-cohort effect among those with higher baseline CD4 counts, or residual confounding by variables not captured in this dataset. A similar phenomenon has been observed in other cohort analyses, in which greater disease severity at baseline paradoxically predicted better engagement with care and virological outcomes, likely mediated through more intensive health care contact. Conversely, patients who present with relatively preserved immunity may be perceived as being at lower risk, receive less frequent monitoring, and may have a more variable pattern of virological suppression over time. These findings reinforce the imperative for sustained, uniform engagement across the care continuum regardless of CD4 cell count at presentation [7,28].

Neither baseline VL change nor CD4 ≥500 cells/µL was an independent predictor of virological outcome in the multivariable model. The near-perfect collinearity between VL Change and baseline VL (Spearman’s *ρ* = 0.971) is consistent with the inherent mathematical relationship between these variables and limits their independent statistical contribution.

Contextualizing our findings against the 95-95-95 targets is instructive. Elgalib et al. [7] estimated that only 55% of Oman’s estimated 2440 PLWH were virally suppressed at the population level, falling substantially short of the third 95% target. The virological suppression rate in our cohort, drawn from the primary national referral center and representing a more treatment-experienced population, was 93.3% (421/451 patients with an available Last VL) at last follow-up, allowing direct benchmarking against this national estimate. The observation that younger patients were at highest risk for non-suppression mirrors the national pattern identified by Elgalib et al., in which the 13-24 age group showed the greatest attrition across the cascade. Closing this age-specific gap will be critical if Oman is to approach the 95-95-95 targets in the coming decade [8].

Several strengths of this study deserve acknowledgment. This is the largest single-center HIV cohort reported from Oman to date, with 454 patients followed over a 13-year period spanning both the introduction of the treat-all policy and the national transition to dolutegravir-based first-line ART, a combination unique in the available Omani literature. Complete virological and immunological laboratory data were available for the great majority of patients, supporting reliable multivariable regression analyses. The use of standardized electronic medical records ensured consistency of data capture across the study period. These features substantially enhance the internal validity and clinical relevance of the findings.

## Limitations

This study has some limitations that should be considered when interpreting the findings. First, as a retrospective, single-center study, the findings may not be fully generalizable to all PLWH in Oman, particularly those managed at smaller regional centers. Second, reliance on electronic medical records meant that data completeness was variable; up to 41 observations were missing for some continuous variables, and analyses were conducted on complete cases without imputation, which may introduce selection bias. Third, the mode of HIV transmission was not available for a substantial proportion of cases, limiting epidemiological characterization. Fourth, treatment adherence data, socioeconomic status, and stigma-related variables, all of which may influence virological outcomes, were not systematically captured in this dataset. Fifth, the study period spanned over a decade (2010-2023), during which ART regimens, monitoring protocols, and treatment guidelines evolved considerably; temporal confounding may therefore influence outcome associations. Sixth, the binary definition of viral suppression (VL <1000 copies/mL) used in the logistic regression model, while clinically common, differs from the more stringent WHO target of <50 copies/mL and may underestimate the prevalence of low-level viraemia. Seventh, model discrimination was assessed only by sensitivity at a single classification threshold; a formal discrimination statistic, such as the area under the receiver operating characteristic curve, was not calculated, and the modest McFadden pseudo-*R²* (0.149) indicates that a substantial proportion of the variance in virological outcome remains unexplained by the measured predictors, consistent with the likely influence of unmeasured factors such as adherence and psychosocial circumstances noted above. Finally, the hypothesis that the paradoxical association between a higher baseline CD4 count and lower odds of viral suppression reflects differences in the intensity of clinical monitoring could not be directly tested, as data on visit frequency and engagement with care were not captured; this interpretation therefore remains speculative and warrants dedicated prospective evaluation.

## Conclusions

This study provides an updated and detailed description of the demographic, clinical, and virological profiles of PLWH receiving ART at the Royal Hospital, Oman. The cohort comprised predominantly male Omani citizens of working age, and most patients presented at an early, asymptomatic WHO clinical stage 1. Heterosexual transmission remained the dominant mode of transmission. HBV and HCV co-infections and TB co-infection were documented in one-quarter of the patients, reinforcing the importance of systematic co-infection screening. Older age at HIV diagnosis was independently associated with a lower likelihood of virological non-suppression, whereas presenting with higher CD4 counts was paradoxically associated with lower odds of viral suppression at last follow-up, likely reflecting differences in the intensity of clinical follow-up. These findings underscore the need for consistent, universal engagement across all disease stages, targeted support for younger patients, and continued strengthening of the HIV care cascade at regional and national levels. Future multicenter, prospective studies incorporating adherence data, quality-of-life measures, and longer-term outcomes will be important to further advance the evidence base for HIV management in Oman and the broader MENA region.

## CRediT authorship contribution statement

FK, HAA, MS and KB conceptualized the project and conducted initial background research; FK, HAA, MS and KB planned and conducted the formal analysis and validation; JAN and AA contributed to data collection and verification; FK, HAA, MS, KB, JAN, AA, and SA interpreted the results; FK, HAA, and SA led the writing (original draft and editing) of the manuscript; and FK, HAA, and SA supervised the project. All authors approved the final article.

## Declaration of competing interest

The authors have no competing interests to declare.
